# Neutrophils in ANCA-associated vasculitis: Mechanisms and implications for management

**DOI:** 10.3389/fphar.2022.957660

**Published:** 2022-09-23

**Authors:** Shangqing Ge, Xingyu Zhu, Qinyao Xu, Junyan Wang, Cheng An, Ying Hu, Fan Yang, Xinyi Wang, Yipin Yang, Shuwen Chen, Ruimin Jin, Haiyan Li, Xinchen Peng, Yue Liu, Junnan Xu, Minhui Zhu, Zongwen Shuai

**Affiliations:** ^1^ Department of Rheumatology and Immunology, The First Affiliated Hospital of Anhui Medical University, Hefei, Anhui, China; ^2^ National Institute of Clinical Drug Trials, The First Affiliated Hospital of Bengbu Medical College, Bengbu, Anhui, China; ^3^ Department of Clinical Medical, The Second Clinical Medical College, Anhui Medical University, Hefei, Anhui, China; ^4^ Department of Cardiovascular Surgery, The First Affiliated Hospital of Anhui Medical University, Hefei, Anhui, China; ^5^ Inflammation and Immune Mediated Diseases Laboratory of Anhui Province, Anhui Institute of Innovative Drugs, School of Pharmacy, Anhui Medical University, Hefei, Anhui, China; ^6^ Department of Clinical Medical, The First Clinical Medical College, Anhui Medical University, Hefei, Anhui, China

**Keywords:** ANCA, aav, neutrophil, biomarker, proteinase 3

## Abstract

Antineutrophil cytoplasmic antibody (ANCA)-associated vasculitis (AAV) is a group of systemic autoimmune diseases, which is typified by inflammatory necrosis predominantly affecting the small vessels and often accompanied by positive ANCA. Clinically, AAV primarily includes microscopic polyangiitis (MPA), granulomatosis with polyangiitis (GPA), and eosinophilic granulomatosis with polyangiitis (EGPA). It has been found that in AAV pathogenesis, both innate and adaptive immunity are related to neutrophil function mutually. Many proteins, such as myeloperoxidase (MPO) and proteinase 3 (PR3), in neutrophil cytoplasm lead to the production of proteins such as MPO-ANCA and PR3-ANCA by activating adaptive immunity. In addition, through the process of neutrophil extracellular trap (NET) formation, activation of an alternative complement pathway and the respiratory burst can stimulate the neutrophils close to vascular endothelial cells and will participate the vessel inflammation. This review aims to reveal the potential mechanisms regulating the association between the neutrophils and various types of AAVs and to emphasize the results of recent findings on these interactions. Moreover, multiple underlying signaling pathways involved in the regulation of neutrophils during AAV processes have also been discussed. The ultimate goal of this review is to identify novel biomarkers and therapeutic targets for AAV management in the future.

## 1 Introduction

Vasculitis is the presence of inflammatory cell infiltration in and around the vessel wall, which accounts for vascular injury, including cellulose deposition, collagen fiber degeneration, and endothelial cell and smooth muscle necrosis. AAV stands for antineutrophil cytoplasmic antibody (ANCA)-associated vasculitis. As one major sub-group of vasculitis, AAV is mainly characterized by inflammation and destruction primarily affecting the small blood vessels (arterioles, capillaries, and venules) and is often accompanied by the presence of circulating ANCA (Geetha and Jefferson 2020; Kitching et al., 2020). It principally consists of granulomatosis with polyangiitis (GPA), microscopic polyangiitis (MPA), and eosinophilic granulomatosis with polyangiitis (EGPA) (Jennette et al., 2013).

Neutrophils are the major components of white blood cells and an important constituent of the host’s innate immune system. Neutrophils, by virtue of their natural functions of chemotaxis, phagocytosis, bactericidal effects, formation of neutrophil extracellular traps (NETs), etc., not only play critical roles in the defense of infectious diseases but also in the process of tissue regeneration (Nauseef and Borregaard, 2014; Papayannopoulos, 2018). Nevertheless, in some circumstances, the activity of neutrophils may be damaging to the host, such as in autoimmune diseases and cancers (van Rees et al., 2016). One of its fascinating functions, which is becoming increasingly well recognized, is that neutrophils could potentially act as a key link between innate and adaptive immunity, especially in AAV (Thieblemont et al., 2016). ANCAs composed of various autoantibodies are the key hallmark of AAV. The autoantigens recognized by ANCAs mainly exist in the plasma of neutrophils but are also found in small amounts in the monocytes. Therefore, the neutrophil plays a vital role in AAV pathogenesis as both the target cell is attacked by autoantibodies (ANCAs) and as the main participating cell regulating the inflammatory process. Among the various autoantigens in the neutrophil cytoplasm, MPO (myeloperoxidase) and PR3 (proteinase 3) have been demonstrated to be most associated with AAV. The autoantibodies identifying them are called MPO-ANCA and PR3-ANCA, respectively. They have been reported to be related to different AAVs to varying degrees (Guchelaar et al., 2021).

Here, by reviewing the regulating role and mechanism of neutrophils in AAVs, our aim is to reveal the potential therapeutic targets and novel biomarkers for AAV diagnosis and prognosis. The literature review might form the basis for the identification of valuable unexplored spots for further study to improve AAV diagnosis, treatment, and prognosis.

## 2 Overview of AAV

AAV disorders are characterized by blood vessel inflammation, endothelial injury, and tissue damage. MPA, GPA (formerly known as Wegener granulomatosis, WG), and EGPA (formerly known as Churg–Strauss syndrome, CSS) are three major kinds of small-vessel vasculitis marked by significant loss of immune tolerance to primary zymoproteins in neutrophil plasma, most commonly myeloperoxidase (MPO) and proteinase 3 (PR3) to produce MPO-ANCA and PR3-ANCA, respectively (Kitching et al., 2020).

Epidemiological research related to AAV presents many complex challenges due to the rarity of the disease, the sort of clear definition to identify the diseases and the differences found in the classification criteria recognized to differentiate the diseases from other disorders in the past. In addition, available data of the various epidemiological studies on AAV suggested that incidence and prevalence might have increased over the past 30 years. For instance, in the past three decades, the annual incidence rate per million of GPA and MPA in the United States increased from 8.6 and 2.9 to 13 and 16, respectively, whereas this incidence rate ascended from 6 and 3 to 34 and 13, respectively, in Germany, and from 6.6 and 2.7 to 15.6 and 6.5, respectively, in Norway. As for the prevalence, the rate per million of GPA increased from 32 to 218 in the United States, from 42 to 210 in Germany, and from 53 to 261 in Norway. The prevalence rate per million of MPA was reported to be 184, 46, and 58.2 in the United States (1996–2015), Germany (2013–2016), and Norway (1999–2013), respectively (Ntatsaki et al., 2010; Berti et al., 2017; Mohammad 2020; Nilsen et al., 2020; Hellmich et al., 2021). Moreover, compared with GPA and MPA, EGPA was found to be the rarest of the three. Its annual incidence rate per million was 1, 1.4, and 4 in Germany (1998–2002), the United Kingdom (2000–2004), and the United States (1996–2015), respectively (Watts et al., 2008; Ntatsaki et al., 2010; Berti et al., 2017), also showing an increasing trend. Many possible explanations for the increase in AAV incidence and prevalence have been proposed. It may be a genuine increase due to various environmental changes, the evolution of classification criteria and the definition, the wider use of the ANCA test to aid proper diagnosis, greater physician awareness through education and training, and greater survival as a result of novel therapeutic advancements (Watts et al., 2022). Moreover, AAV epidemic characteristics were reported to vary in different geographical areas throughout the world. It was more prevalent in white and Asian people but less prevalent in African-Americans (Geetha and Jefferson, 2020; Watts et al., 2022). In Japan, the annual incidence of MPA with renal complication was 14.8/million, and MPA with positive MPO-ANCA was the most common in AAV, whereas in the United Kingdom, GPA with positive PR3-ANCA was the most common AAV. Furthermore, clinical characteristics of renal vasculitis second to AAV were also reported to be significantly different between Japan and the United Kingdom (Fujimoto et al., 2006; Watts et al., 2008). AAV in China seemed to share AAV epidemic and clinical characteristics with cases found in Japan (Liu et al., 2008).

Though the exact cause of AAV remains an enigma, it is widely accepted today that the interplay of infectious, genetic susceptibility, and environmental factors could account for AAV onset by immune intolerance to autoantigens in neutrophil cytoplasm (Salvador 2020). Autoantibodies, mainly including MPO-ANCA and PR3-ANCA, not only are current practical diagnostic biomarkers (Grayson et al., 2022; Robson et al., 2022; Suppiah et al., 2022) but also play important pathogenic roles in the mechanism of AAV (Hutton et al., 2017; Massicotte-Azarniouch et al., 2022). Moreover, the formation of NETs, activation of an alternative complement pathway (ACP), and immune imbalance of T-cell subgroups specific to the same autoantigens with ANCA might all be involved in the pathogenesis of this vasculitis (Brilland et al., 2020; Wang et al., 2020; Negreros and Flores-Suarez 2021). The various clinical manifestations of AAV often include a wide range of symptoms and signs due to the disorders of general status and damage to the renal, respiratory, nervous, and other systems (Hunter et al., 2020). Moreover, some of the injury complications may be fatal to life, such as acute renal failure and alveolar hemorrhage (Quartuccio et al., 2020; Oristrell et al., 2021).

AAV treatment development is primarily based on our understanding of its pathogenesis. As a group of autoimmune diseases, glucocorticoids (GC) and other immunosuppressants have been employed as the classical drugs for the management of AAV (Yates et al., 2016; Chung et al., 2021). The dose of GC used depends on the patient’s clinical condition. Cyclophosphamide (CTX) has been used as the first choice of immunosuppressants to induce AAV remission. Moreover, other immunosuppressants, such as azathioprine (AZP), mycophenolate mofetil (MMF), and methotrexate (MTX), are often selected to maintain the remission (Yates et al., 2016; Chung et al., 2021). ANCA are characterized by autoantibodies produced by plasma cells derived from B cells which play the primary pathogenic role in AAV, and thus, targeted deletion of B lymphocytes to decrease the level of ANCA by the monoclonal antibody (Rituximab) against CD20 has been proved to be very effective in AAV treatment (Raffray and Guillevin 2020). In fact, Rituximab has been recommended to induce and maintain the remission of AAV in clinical settings (Yates et al., 2016; Chung et al., 2021). Its clinical efficacy is equal to or even better than that of CTX (Charles et al., 2020; Tieu et al., 2020). In addition, patients diagnosed with AAV in critical conditions (such as alveolar hemorrhage and rapidly progressive nephritis) can be treated by plasmapheresis, which can effectively remove ANCA and reduce the levels of various proinflammatory cytokines directly and rapidly (Yates et al., 2016), although the long-term efficacy of this treatment seems to be controversial recently (Walsh et al., 2020; De Vriese and Fervenza 2021). The phase III clinical trials on the effectiveness and safety of Avacopan in the treatment of AAV have been lately reported to be successful (Jayne et al., 2021). Avacopan is the first oral C5a (fragment a of the fifth complement) receptor inhibitor employed to block the binding of this receptor on the neutrophil with C5a because it has been established that C5a derived from the activation of ACP plays a key role in the pathogenesis of AAV (Bekker et al., 2016; Jayne et al., 2017). However, without appropriate treatment in time, the prognosis of AAV could be very poor, and its death rate was roughly 60% in half a year and 80% in 1 year (Booth et al., 2003). Even under the available treatment today, the 5-year survival for GPA, MPA, and CSS was 74–91%, 45–76%, and 60–97%, respectively (Mukhtyar et al., 2008), and the average mortality of AAV has still increased by 2.7-fold compared with the general population (Tan et al., 2017).

As outlined previously, although some of the pathogenesis, clinical manifestations, and treatments are common between GPA, MPA, and EGPA, the main vasculitis in AAV has its own characteristics due to the diverse roles of neutrophils, which will be briefly described as follows.

Many studies have shown that ANCA can exhibit distinct pathogenic functions in ANCA vasculitis (Hutton et al., 2017). Neutrophils are the main mediators of vessel injury. In response to infection or inflammation, neutrophils are exposed to different inflammatory cytokines (tumor necrosis factor-α and interleukin-1), lipopolysaccharide, or complement C5a and become primed with the movement of MPO and PR3 from the primary granules to the neutrophil surface. In this primed state, ANCAs can bind to these autoantigens on the cell surface, thus resulting in robust cellular activation.

## 3 Overview of neutrophils

Neutrophils are a homogenous population of cells with antimicrobial capabilities such as phagocytosis, degranulation, and the formation of NETs (Silvestre-Roig et al., 2019). It has been reported that by activating many effector pathways, neutrophils can not only help the host defense against pathogens but also clear aging, damaged, and necrotic tissues (Nauseef and Borregaard, 2014; van Rees et al., 2016). Under normal circumstances, neutrophils are in an inactive state and move slowly in the direction of the peripheral blood circulation. Once the body is stimulated to enter into an inflammatory state, neutrophils will be activated, released quickly from the neutrophil storage pool, and stimulated to undergo proliferation in the bone marrow. Neutrophils activated in the peripheral circulation will show a tendency to migrate to the injury site. One of the most important potentials of the neutrophil in this movement is chemotaxis for neutrophil migration, which involves two related capabilities, namely, mobility and directionality. The migration process of a neutrophil typically involves rolling, activation, and adhesion, all of which occur before cross endothelial cell movement. This process is the result of the interaction between the different receptors expressed on neutrophils and ligands expressed on the vascular endothelium (Nauseef and Borregaard 2014). Upon migration to the inflammatory location, neutrophils can devour foreign bodies including degenerated cells and pathogenic microorganisms through chemotaxis, conditioning, swallowing, and sterilization. In addition, neutrophils activated can gather and increase their numbers, change their shapes, release their NETs, practice their respiratory burst, degranulate their superoxide and lysosomal enzymes, and thereby effectively eliminate intrusive microbes, thereby executing their physiologic functions to protect the organism by clearing invading pathogens and aging necrotic tissues (Nauseef and Borregaard, 2014; van Rees et al., 2016).

However, under certain conditions, extensive damage caused by activated neutrophils can lead to some disorders, even autoimmune diseases (Thieblemont et al., 2016). One typical instance of such an autoimmune disease is AAV. Neutrophil cytoplasm contains a large number of different proteases, among which PR3 and MPO are the most important in AAV. After being activated by inducing factors such as inflammatory cytokines (tumor necrosis factor-α and interleukin-1), lipopolysaccharide or complement C5a, neutrophils are primed with the movement of MPO and PR3 from the granules in its cytoplasm to its membrane surface. Neutrophils can be further activated by ANCA and C5a and then they can respectively combine with specific antigens (PR3 and MPO) and the C5a receptor expressed on the neutrophil surface. Activated neutrophils can then attach to vascular endothelial cells and can be transported to the vascular wall to accumulate, produce reactive oxygen species free radicals, and finally elicit cell apoptosis and tissue lesions, resulting in vascular endothelial inflammatory destruction and substantial tissue damage. Endothelial injury can lead to the leakage of serum proteins and coagulation factors, causing vascular wall cellulose necrosis and even thrombosis (Al-Hussain et al., 2017; Brilland et al., 2020).

## 4 Functional role of neutrophils in AAV

Neutrophils can play diverse key roles in the pathogenesis of MPA, GPA, and EGPA. It has been reported that compared with neutrophils in the resting state, neutrophils activated by pro-inflammatory factors such as ANCA and C5a can release more pro-inflammatory factors to further activate themselves, activate ACP to produce additional C5a, and promote degranulation and NET formation to discharge more MPO and PR3 from neutrophils. These events can benefit adaptive immunity to produce more MPO-ANCA and PR3-ANCA (Sangaletti et al., 2012; Geetha and Jefferson 2020), thus forming a vicious cycle to deteriorate AAV (Table 1). In addition, eosinophils activated in EGPA can also participate in EGPA pathogenesis by releasing eosinophil cationic protein (ECP), eosinophil-derived neurotoxin (EDN), eosinophil peroxidase (EPO), and others (Gioffredi et al., 2014; Furuta et al., 2019), which could specifically contribute to the distinct clinical features of EPGA (Table 1). These neutrophil-related pathogeneses can result in the clinical characteristics of specific forms of AAV via the respective immune mechanism (Table 2). ANCAs can not only serve as valuable biomarkers for identifying the different subtypes of AAV (GPA, MPA, and EGPA) (Grayson et al., 2022; Robson et al., 2022; Suppiah et al., 2022) but also might be related to different clinical damages (Table 2). In fact, the potential roles of neutrophils in various AAVs are getting increasing attention and these will remain one of the hotspots worthy of further research in the future. The roles of neutrophils in the pathogenesis of different AAVs have been summarized as follows.

**TABLE 1:** Neutrophils/eosinophils and major inflammatory substances involved in the pathogenesis of ANCA-associated vasculitis (AAV**).**

Cell type	Cell state	Related inflammatory substances	Reference
Neutrophil	Resting state	TNF-α, IL-6, IL-18, IL-2Rα (CD25), C5a, G-CSF, GM-CSF, HMGB1, MIF	(PMID: 25841802
			PMID: 27544048
			PMID: 10792393
			PMID: 26410887
			PMID: 22975753
			PMID: 19594951
			PMID: 25889374
			PMID: 24648606)
Neutrophil	Active state	C3a, IL-10, IL-17A, IL23, IL-32, C3bBbP, MIF	(PMID: 20491791
			PMID: 18799068
			PMID: 27544048
			PMID: 26410887
			PMID: 31216309
			PMID: 12124874
			PMID: 31922056)
Eosinophil	Active state	MBP, ECP, EPO, EDN	(PMID: 31266709
			PMID: 25404930)

**TABLE 2:** Main immune factors involved and clinical manifestations of the three major kinds of ANCA-associated vasculitis (AAV).

Classification of AAV	Main immune factors involved	Clinical manifestations	Reference
MPA	Anti-MPO antibody (positive in 60% of patients)		PMID: 31358311
	Anti-PR3 antibody (positive in 30% of patients)	Rapidly progressive renal failure (necrotizing and crescentic glomerulonephritis)	PMID: 22323643 PMID: 30404112
	Anti-LAMP-2 antibody	Chronic kidney injury (eventually leading to end-stage renal disease)	PMID: 20656070)
	Anti-MPO-specific splenocyte	Lung injury	PMID: 29887327
GPA	Anti-PR3 antibody (positive in 75% of patients)	Recurrent sinusitis, crusty rhinorrhea, pulmonary nodules	PMID: 25149391
	Anti-MPO antibody (positive in 20% of patients)	Rapidly progressive necrotizing glomerulonephritis with extra-capillary crescent	PMID: 31358311
	Anti-LAMP-2 antibody		PMID: 24485158)
EGPA	Anti-MPO antibody (positive in 45% of patients)	Allergic phase: characterized by asthma, allergic rhinitis, and sinusitis	PMID: 31266709 PMID: 31358311
	Anti-PR3 antibody (positive in 5% of patients)	Eosinophilic phase: characterized by eosinophilic infiltration (eg. lung, heart, and gastrointestinal system)	PMID: 25404930)
	Eosinophil cytotoxic granule protein, ECP, EPO, EDN	Vasculitic phase: purpura, peripheral neuropathy, and systemic symptoms are the main features	
	IL-5, lipid mediator		

### 4.1 Microscopic polyangiitis

**FIGURE 1:**
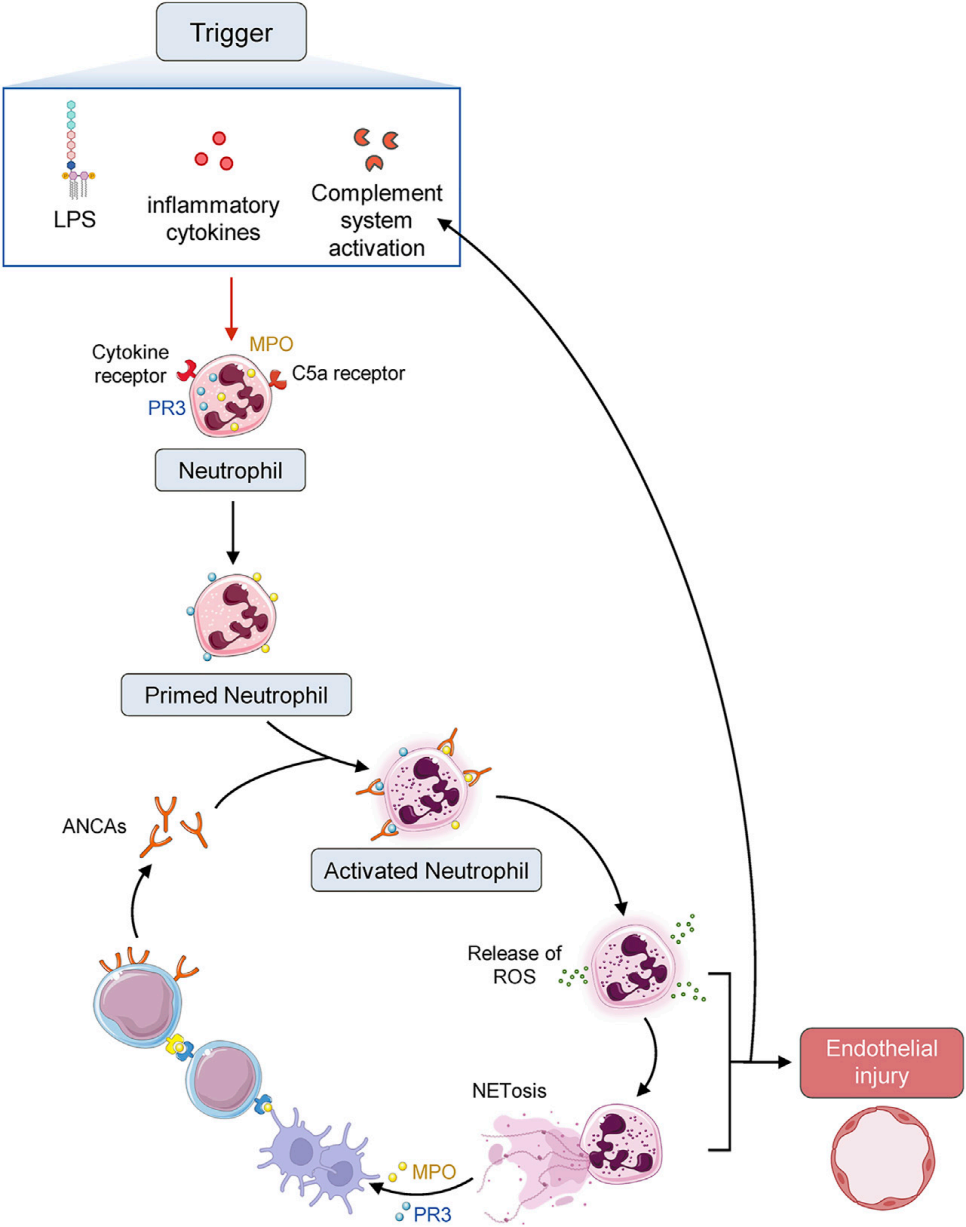
Diagrammatic sketch of AAV pathogenesis. ANCA autoantigens proteinase 3 (PR3) and myeloperoxidase (MPO) identified by ANCA are normally sequestered in the primary granules of neutrophils. Infection or other environmental stimuli result in neutrophil priming, with the movement of PR3 and MPO to the cell surface and activate the neutrophils, which adhere to the vascular endothelium. Neutrophil degranulation leads to the release of reactive oxygen species (ROS), proteases, and neutrophil extracellular traps (NETs), damaging the endothelium. Chemokines and tissue deposition of PR3 and MPO lead to the recruitment of autoreactive T cells and monocytes, augmenting tissue injury.

MPA is an inflammatory illness characterized by systemic vasculitis that mainly affects small-caliber blood vessels and is related to ANCA (Chung and Seo 2010). In terms of MPA pathogenesis, the current accepted views suggest that the pathogenic process of MPA is composed of two phases, with neutrophils serving as the primary executant. The first stage involves priming neutrophils by exposing them to modest amounts of pro-inflammatory cytokines such as IL-1 (interleukin-1) and TNF-α (tumor necrosis factor-α) (Kallenberg et al., 2006). This causes neutrophils expressing MPO and PR3 on their surface to stick to the vascular endothelial cells. In addition to the pro-inflammatory cytokines, lipopolysaccharide and C5a can also promote the migration of MPO and PR3 from the primary granules present in the cytoplasm of neutrophils to their surface. C5a in AAV is mainly from the cascade of ACP activation (Xiao et al., 2007; Ohlsson et al., 2019; Brilland et al., 2020). It is very important in the regulation of innate immune response by C5a receptor 1 (C5aR1) and C5a receptor 2 (C5aR2) (Pandey et al., 2020). ANCAs can specifically bind to their autoantigens (MPO and PR3) on the neutrophil surface in this primed condition, thereby resulting in powerful cellular activation (Kettritz 2012; Geetha and Jefferson 2020). In the second phase, neutrophils can be activated by the interactions between ANCA and either its specific antigens or the Fc receptor on neutrophils (Guilpain et al., 2007; Pankhurst et al., 2011). Activated neutrophils can substantially alter the expression of adhesion molecules and adhere to the endothelium of the vascular system. The increased production of reactive oxygen species and proteases by neutrophil degranulation can cause extensive tissue damage (Wang C. et al., 2017). Activated neutrophils also undergo a process known as NETosis, in which NETs are extruded from the cells, encasing MPO, PR3, and complement components in a chromatin web (Yoshida et al., 2016). NETs can also cause endothelial damage and transport MPO/PR3 to vascular endothelium and dendritic cells for antigen presentation, thereby activating the ACP (O'Sullivan and Holdsworth, 2021; Sangaletti et al., 2012). The various chemokines and PR3 and MPO deposited in the tissue result in the recruitment of autoreactive T cells and monocytes, which can effectively exacerbate and maintain tissue damage (Gapud et al., 2017; Geetha and Jefferson 2020). Several animal experiments have demonstrated that MPO-ANCA might be a directly pathogenic autoantibody involved in MPA pathogenesis (Xiao et al., 2002; Little et al., 2005; Wang Q. et al., 2017), thus indicating that MPO-ANCA could be sufficient to cause pulmonary capillaritis and glomerulonephritis in certain biological environments (Figure 1). Moreover, there was a report that a mother with positive MPO-ANCA gave birth to her neonate who manifested fetal pulmonary hemorrhage and renal failure, and supporting MPO-ANCA from the mother may be able to result in MAP (mean airway pressure) damage in the newborn (Schlieben et al., 2005). However, a later case report revealed that MPO-ANCA transferred through the placenta was not enough to cause neonate illness (Silva et al., 2009). Other co-factors, including genetic predisposition, are likely required for the development of vasculitis caused by ANCA (Chung and Seo 2010). In addition, there are a considerable proportion of MPA patients whose ANCA was always found to be negative (Falk and Hoffman 2007; Geetha and Jefferson 2020). Furthermore, the titer of MPO-ANCA does not always correspond with the disease activity of MPA (Tomasson et al., 2012). These findings suggested that ANCA was not essential for the pathogenesis of all MPA, or that many other mechanisms might contribute to the same clinical manifestations (Chung and Seo 2010).

### 4.2 Granulomatosis with polyangiitis

GPA (previously Wegener’s granulomatosis) is an autoimmune small vessel vasculitis that has been strongly linked to antineutrophil cytoplasmic antibodies (ANCA). Systemic necrotizing vasculitis, necrotizing granulomatous inflammation, and necrotizing glomerulonephritis are all synonyms of this illness (Lutalo and D'Cruz 2014). GPA can affect both men and women between the ages of 45 and 60, but it is uncommon in black people. Recurrent sinusitis, crusting rhinorrhea, pulmonary nodules, progressive necrotizing glomerulonephritis with extra-capillary crescent, and other related disorders can all be caused by GPA in its complete form, which includes symptoms contributed to ENT (ears, nose, and throat), lung, and kidney involvement. The presence of c-ANCAs in approximately 90% of systemic forms and 50% of localized forms directed against PR3 in most instances is one of the main hallmarks of GPA (Puéchal 2020). GPA exhibits a complicated immunopathogenesis that includes the production of ANCA against PR3 in roughly 80% of GPA patients and MPO in approximately 10% of GPA patients (Flint et al., 2010). Antibodies against the lysosome-associated membrane protein-2 (LAMP-2) can potentially contribute to the pathophysiology of GPA by affecting the molecular mimicry pathway (Flint et al., 2010). Currently, environmental or viral triggers in a genetically predisposed person who displays tolerance to the ANCA self-antigen have been assumed to be the main immunopathogenesis of GPA (Lutalo and D'Cruz 2014).

In genetically predisposed people, noxious stimuli can cause an inflammatory response that includes the release of pro-inflammatory cytokines and the formation of ANCA. The recurrent, relapsing pattern of GPA might be connected to chronic colonization of nasal passages with *Staphylococcus aureus*, a common microbe involved in the etiology of the illness. This Gram-positive bacteria produce super-antigens that can activate both B and T cells, and it can also generate AAV by modulating the molecular mimicry pathway (Flint et al., 2010). When compared to healthy persons, patients with GPA often exhibit significantly higher levels of B-lymphocyte stimulator factors, such as B-cell activation factor (BAFF), and a higher proportion of T follicular helper cells (TFH) (Abdulahad et al., 2013; Watts et al., 2022). This might explain the observation of why GPA patients display a higher rate of self-reactive B cells. These self-reactive B lymphocytes can develop into long-lived plasma cells that release ANCA, a pathogenic autoantibody linked to GPA that can interact with PR3 found on the surface of neutrophils and monocytes. When neutrophils and monocytes are exposed to ANCA, they can produce and release many mediators, including reactive oxygen species, proteases, cytokines, and neutrophil extracellular trap products (NET-derived products) (Flint et al., 2010). Toll-like receptors (TLRs) can be triggered by NET-derived products, and the production of interferon-α (IFN-α) can inhibit the functions of T-regulatory cells (Flint et al., 2010). The membrane attack complex (C5b6789 MAC) is formed when the alternative complement signal pathway is activated, which can then enhance ANCA-related neutrophil activation, inflammation, and tissue damage (Flint et al., 2010). These pro-inflammatory signaling pathways can cause necrotizing systemic vasculitis, necrotizing glomerulonephritis, and granulomatous inflammation of the airways, all of which are regarded as key hallmarks of GPA.

### 4.3 Eosinophilic granulomatosis with polyangiitis

EGPA, formerly known as Churg–Strauss syndrome, is a systemic necrotizing vasculitis of small and medium-sized arteries, which is primarily characterized by asthma and blood eosinophilia. EGPA is a disorder that can affect multiple organs including the skin, lungs, and peripheral nerves in those who already have been diagnosed with asthma (Nguyen and Guillevin 2018). In 2012, the Chapel Hill Consensus Conference replaced the old eponym (Churg–Strauss syndrome) with EGPA and classified the condition as an AAV disease (Jennette et al., 2013). Furthermore, EGPA mainly differs from GPA and MPA in terms of ANCA expression. ANCA has been identified in just 30–40% of EGPA patients (Sinico et al., 2005; Comarmond et al., 2013; Moosig et al., 2013), but in 70–90% of GPA and MPA patients (Villiger and Guillevin, 2010; Comarmond et al., 2013; Moosig et al., 2013; Comarmond and Cacoub, 2014). In addition, a few EGPA patients who do not display any histologic signs of vasculitis might be affected by this condition. In fact, these individuals might be categorized as having hyper-eosinophilic syndromes (HES) or suffering from eosinophil lung disease (Valent et al., 2012).

EGPA is associated with diverse clinical and pathological patterns and arises because of different mechanisms of immunological dysregulations. CD4^+^ T cell is an important immune cell in the human immune system and its multifaceted immune functions are very valuable for analyses of its potential roles in GPA mechanisms. Both Th1 and Th2 are important subgroups of CD4^+^ T cells, and their signaling pathways are active with eosinophils being responsible for the majority of tissue damage (Trivioli et al., 2020). CD4^+^ T cells derived from peripheral blood or broncho-alveolar lavage have been reported to produce more Th2-related cytokines, including IL-5, IL-10, and IL-13, which can effectively promote eosinophil maturation in the bone marrow and play a vital role in peripheral activation (Kiene et al., 2001). The success of therapy based on IL-5 antagonism underpins the importance of this signaling route. Furthermore, CD4^+^ T cells can shield eosinophils from apoptosis and aid them to survive longer (Tsurikisawa et al., 2013). However, recent studies have revealed that eosinophil proliferation triggered by tyrosine-kinase signaling pathways might potentially be implicated (Emmi et al., 2015). Endothelial cells can emit eotaxin-3, which can cause eosinophils to infiltrate into the tissues and release cytotoxic granule proteins such as ECP and major basic proteins (MBP) (Polzer et al., 2008). In addition, ECP might promote cell death and present antigens to Th cells, thus perpetuating a vicious cycle (Terrier et al., 2010). Interferon can mediate granuloma development, which is a strategy to keep the harmful compounds secreted by eosinophils at bay. Neutrophils are commonly observed in vascular lesions. It is worth indicating that the levels of IL-17, which can promote recruitment and activation of neutrophils are significantly increased in active EGPA (Jakiela et al., 2011). B cells may also potentially play a role, according to the results of CD20^+^ B-cell depletion. It is an important progenitor of ANCA-producing plasma cell, and thus, might deliver antigens to activate Th cells. Th2-related cytokines, in turn, can boost the isotopic shift toward IgE and IgG4. However, the latter are ineffective at activating complement signaling pathways, and their functions are relatively unknown. Furthermore, the various etiological and triggering factors in EGPA have been poorly understood. Infectious agents and immunizations have not yet been recognized as triggers of illness. Immunogenetic variables can confer vulnerability to EGPA, according to candidate gene association studies (Vaglio et al., 2007). Furthermore, the IL-10.2 haplotype of the IL-10 gene promoter is predominantly linked with higher production of IL-10, a Th2-related cytokine, in ANCA-negative individuals (Wieczorek et al., 2008). The genetic susceptibility patterns of EGPA and the related sub-phenotypes are expected to be clarified by ongoing genome-wide association studies (Trivioli et al., 2020).

## 5 Regulatory mechanisms in AAV

The onset, development, prognosis, and other processes of AAV are known to involve complicated molecular signaling pathways, and our present understanding of these mechanisms is insufficient, thus resulting in a poor cure rate of AAV. Even so, the mechanisms of signaling pathways implicated in modulating the neutrophils in AAV have been better understood, which will be summarized in the following sections.

### 5.1 MAPK signaling pathway in the pathogenesis of AAV

The mitogen-activated protein kinases’ (MAPK) signaling system plays a key role in the regulation of cell proliferation, differentiation, apoptosis, invasion, and migration throughout the life span of the cell cycle. The MAPK cascade is mainly activated when neutrophils are exposed to numerous stimuli such as UV irradiation, growth factors, and cytokines. Protein phosphorylation is the fundamental, most prevalent, and important mechanism by which protein viability and function can be effectively regulated and controlled. Protein phosphorylation occurs primarily on two amino acids, serine (including threonine) and tyrosine (Hunter 2014). The activation of human neutrophils by ANCA relies heavily on the phosphorylation of tyrosine residues, particularly MAPK (Hao et al., 2012). MAPKs are triggered by upstream dual-specificity kinases phosphorylating threonine and tyrosine residues and they serve as powerful inflammatory signaling pathways (Hao et al., 2012).

**FIGURE 2:**
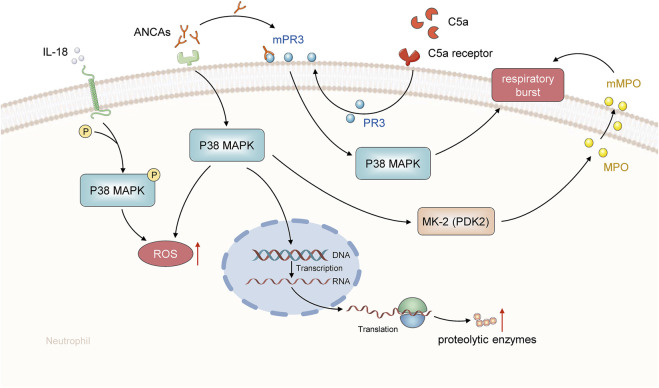
MAPK signaling pathway in the pathogenesis of AAV. ANCA activates the p38MAPK-mediated signaling pathway in human neutrophils. This signal cascade is responsible for the translocation of ANCA-specific antigens from the cytoplasmic granulosa to the neutrophil surface. This translocation enables these antigens to bind with their specific autoantibodies. IL-18 initiates ANCA-induced production of neutrophil superoxide by phosphorylating p38MAPK.

Complement activation can also play a crucial role in the pathogenesis of AAV, according to a recent study (Xiao et al., 2007). C5a is one of the most powerful inflammatory peptides among the complement activation products, having a wide range of actions. C5a acts as a potent neutrophil chemoattractant and can display chemotactic action in both monocytes and macrophages (Guo and Ward 2005). C5a works by primarily binding to the high-affinity C5a receptor on neutrophils. The activation of p38MAPK, extracellular signal-regulated kinase (ERK), and phosphoinositide 3-kinase (PI3K) is possibly involved in the translocation of ANCA antigens and C5a-induced neutrophil activation by ANCA. To further understand how ANCAs can potentially stimulate neutrophils, several experiments to identify the signaling pathways have been conducted (Schreiber et al., 2010; von Vietinghoff et al., 2007; Hao et al., 2012). The most noteworthy discovery in these experimental studies was that the p38MAPK, ERK, and PI3K signaling pathways have been implicated in ANCA-mediated modulation in C5a-primed neutrophils. Moreover, targeted suppression of the membrane expression of the ANCA-specific antigen by employing p38MAPK, ERK, and PI3K inhibitors could effectively prevent C5a-primed neutrophils from undergoing respiratory burst induced by ANCA (Hao et al., 2012). In addition, only p38MAPK and ERK have been reported to be involved in human neutrophils, despite the fact that three MAPK signaling pathways have been identified (Hao et al., 2012). Moreover, accumulating pieces of evidence indicate that tyrosine phosphorylation and p38MAPK regulation on the translocation of the ANCA antigen to the cell surface primed with TNF-α might be the probable mechanisms of ANCA-stimulated respiratory burst in provoked neutrophils (Kettritz 2012), (Figure 2).

### 5.2 PI3K signaling pathway in the pathogenesis of AAV

The phosphatidylinositol 3-kinase (PI3K) family can actively participate in a variety of signaling pathways and is involved in regulating cell proliferation, differentiation, apoptosis, and glucose transport. The PI3K-mediated signal transduction system has been also found to be indispensable in the pathogenesis of AAV. For instance, under *in vitro* settings, ANCA can activate human polymorphonuclear neutrophils (PMN) that have been primed with TNF-α (von Vietinghoff et al., 2007). Protein-serine/threonine kinase Akt and PI3K have been reported to be involved in the modulation of the phagocyte respiratory burst, and PI3K can control the ANCA-induced respiratory burst. Phosphatidylinositol-3,4,5-triphosphate (PIP3) and phosphatidylinositol-4-diphosphate (PIP4) are mainly produced by phosphatidylinositol 4,5-bisphosphate (PIP2). Both the products are required for the recruitment of serine/threonine kinase Akt to the plasma membrane, where they may be phosphorylated by phosphoinositide-dependent kinase-1 (PDK1) at threonine 308 (T308) and phosphoinositide-dependent kinase-2 (PDK2) at S473 by PDK1. In addition, PI3K products or the p38MAPK substrate MK-2 can both activate Akt (von Vietinghoffet al., 2007). The Akt signaling module has been investigated in detail and it was discovered that Akt, p21 activated kinase 1 (PAK1), heat-shock protein 27 (HSP27), and Ras-related C3 botulinum toxin substrate 1 (Rac1) reside in the complex in the cytoplasm of resting PMNs, and that TNF-α stimulation enhanced PAK1 interaction with Akt (von Vietinghoff et al., 2007). Moreover, changes in the cytoskeleton are required for stimulating the PMN respiratory burst and the translocation of ANCA antigens (Matsushima et al., 2014). In human PMN, Akt, p38MAPK, MK-2, and HSP27 can form a signaling complex, and cellular activity can change based on the composition of the complex. The interaction of Akt with cytoskeleton-regulating proteins might represent a site of convergence of the various signaling pathways implicated in the response to TNF-α and ANCA (von Vietinghoff et al., 2007).

**FIGURE 3:**
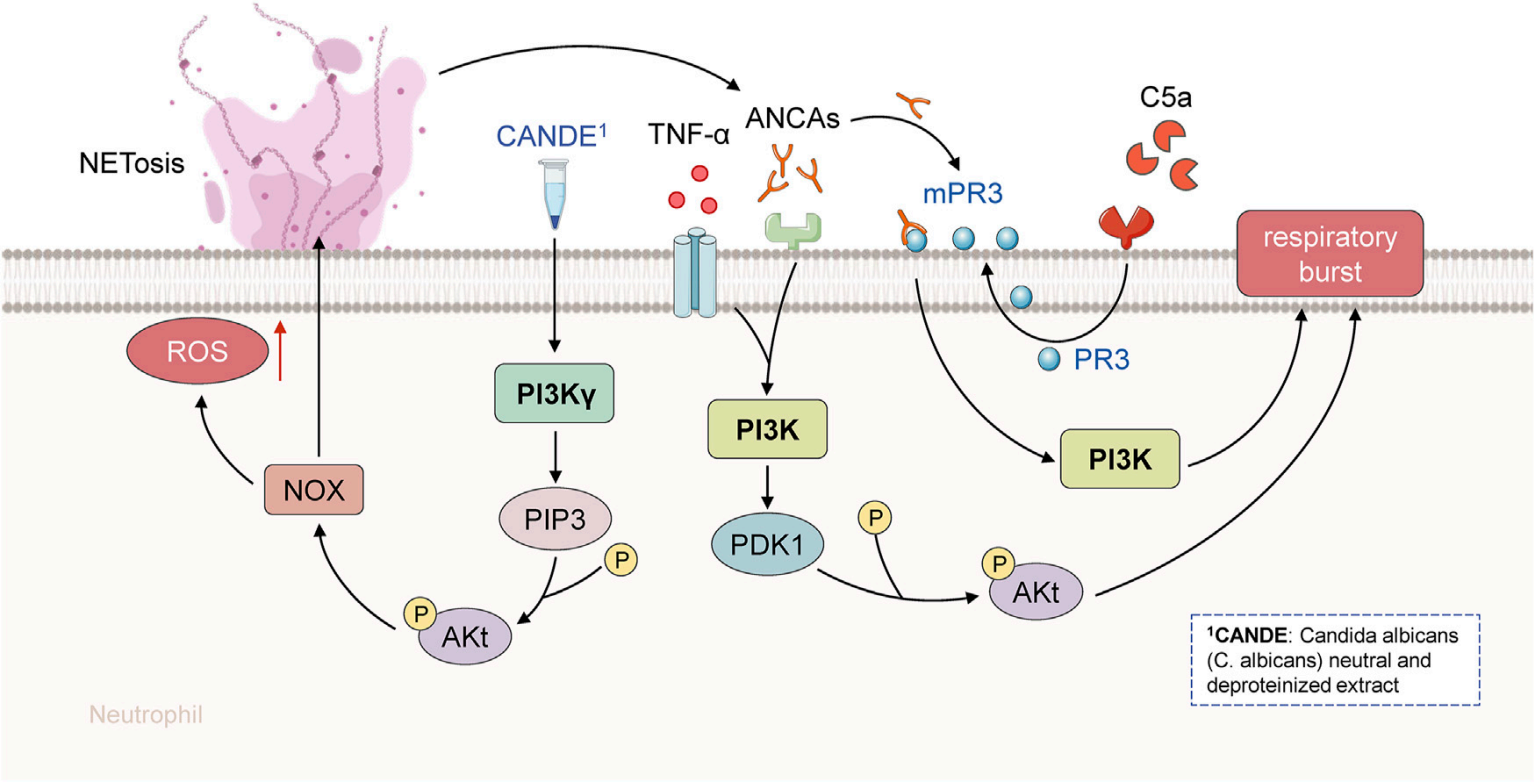
PI3K signaling pathway in the pathogenesis of AAV. ANCA and TNF-α activated human polymorphonuclear neutrophils. PI3K was activated during TNF-α initiation. PI3K and Akt are involved in the control of phagocyte respiratory burst, while PI3K can control ANCA-induced respiratory burst. Akt can be activated and phosphorylated by the product of PI3K or p38MAPK substrate MK-2.

In conclusion, PI3K and p38MAPK are both reported to be involved in the ANCA-induced respiratory burst. TNF-α induced translocation of ANCA is mainly aided by p38MAPK activation, whereas PI3K is required for ANCA-induced respiratory burst. Because PI3K can operate as an upstream activator of p38MAPK, it appears that PI3K activation is essential for the activation of human neutrophils by TNF-α and ANCA (von Vietinghoff et al., 2007). In fact, all cellular signaling pathways involved in the regulation of neutrophils might play a crucial role in the occurrence, development, and multiple clinical damages of AAVs, thus implying that neutrophils can serve as a potential target for AAV therapy and as a key factor determining the prognosis of AAVs (Figure 3).

## 6 Clinical potential of the role of neutrophils in AAV

At present, the clinical treatment for AAV, irrespective of the stage of diagnosis, is composed of two phases, namely, induced remission and maintain remission therapy (Yates et al., 2016; Chung et al., 2021). Therapeutic regimes in both of these phases are directly or indirectly related to neutrophils as the autoimmune disorder leading to AAV primarily targets the autoantigens in neutrophils, and as discussed previously, neutrophils can play a critical role in AAV pathogenesis. Moreover, in approved clinical treatment for AAVs, in addition to GC and conventional immunosuppressants such as CTX, MTX, MMF, and AZP, the monoclonal antibodies against CD20 (rituximab and RTX) and the blocker of C5aR1 (Avacopan) are conspicuous new drugs (Yates et al., 2016; Chung et al., 2021; Jayne et al., 2021). For instance, RTX is a biological agent initially developed for targeting B-cell lymphoma. It can significantly decrease the source of pathogenic autoantibody ANCA by deleting B cells in AAV patients. Accumulating pieces of evidence based on clinical research have demonstrated that RTX is relatively safe and effective for AAV therapy. It tends to be used instead of CTX in the treatment for induced remission of active AAV, especially for induced remission of refractory and recurrent AAV as CTX and RTX have similar effects and CTX has been related to higher rates of infection (Chung et al., 2021; Thomas et al., 2021). Avacopan is the first oral C5aR antagonist developed to block the activation of neutrophils by C5a. Many prior clinical studies have shown that Avacopan could markedly reduce or even replace GC in the treatment of AAV, which clearly demonstrates its booming prospect in management of AAV (Jayne et al., 2021). Therefore, Avavcopan has been approved for the treatment of ANCA-related diseases (ANCA vasculitis and nephritis) by the FDA on 8 October 2021, soon after its successful results in phase III clinical trials were obtained.

In addition to AAV treatment, immune indicators related to neutrophil autoimmunity can also be employed to predict AAV relapse early enough to avoid substantial organ damage and enhance survival. It has been reported that ANCA- and B-cell status could be predictive of the majority of relapses in a large cohort of AAV patients following remission-induction therapy with RTX, and their absence can potentially indicate highly predicted relapse-free status. Relapses occurred in 96% of PR3-ANCA-positive individuals with persistent or reappearing PR3-ANCAs and 81% with B-cell repopulation. All the relapses in MPO-ANCA-positive individuals were mainly limited to those with persistent MPO-ANCAs and B-cell repopulation. Thus, it can be concluded that the majority of relapses were predicted by ANCA and B-cell status, and their absence could accurately predict relapse-free status. Therefore, the application of ANCA and B-cell surveillance to aid the therapeutic decision-making in AAV patients treated with RTX might help to avoid relapses (Tieu et al., 2020; van Dam et al., 2021).

The aforementioned studies have clearly suggested that immunity related to neutrophils might provide very valuable utilities in AAV treatment and prognosis. However, the precise processes involved remain unknown currently, thereby necessitating more investigation and proper confirmation to determine the optimal therapeutic potential related to neutrophils in AAV.

## 7 Future expectations

With more bench and clinical in-depth studies, the important role of neutrophils in AAV pathogenesis is continuously evolving and has been better understood. Neutrophils are predicted to represent one of the novel targets for AAV therapy. Although the current treatment methods stated previously have been significantly improved, they still have exhibited limited effects in significantly improving the diagnosis and survival rate of AAVs. To develop the potential of neutrophils in the future treatment of AAV, further investigations should mainly focus on NETs, receptors, and signal transmission pathways. In addition, the efficacy and prognostic effects of the conventional treatment, such as plasma exchange (PLEX), need to be further confirmed.

As discussed in the previous section, NETs can play a characteristic role in the pathogenesis of AAV, thereby contributing to innate immunity and adaptive immunity in AAV (Sangaletti et al., 2012; Yoshida et al., 2016; Papayannopoulos, 2018; Negreros and Flores-Suarez, 2021). To prevent the formation of NETs, an important strategy can be the identification of novel inhibitors of peptidyl arginase deiminases, which is a key enzyme for NET generation. Another way could be to investigate the potential inhibitors of neutrophil elastase (NE), which may act in conjunction with Gasdermin D synergistically to break down the nuclear envelope and the outer cell membrane during NET production (Sollberger et al., 2018). Moreover, to facilitate the destruction of the already formed NETs, deoxyribonucleases (DNase), especially DNase I (Schreiber et al., 2017), might be another important therapeutic approach worth further exploring for the treatment of AAV (O'Sullivan and Holdsworth 2021).

Furthermore, for the maintenance of innate immune responses, rapid neutrophil recruitment to areas of inflammation remains critical (Etzioni 2009). It has been found that G-protein-coupled receptor (GPR35)-mediated neutrophil recruitment to inflamed tissue is eliminated in mice lacking 5-hydroxyin doleacetic acid (5-HIAA). This finding identified 5-HIAA as a GPR35 ligand and neutrophil chemoattractant, thereby implying a possible role for 5-HIAA generated by platelets and mast cells in cell recruitment to inflammatory areas and bacterial clearance (De Giovanni et al., 2022). GPR35 has been also linked to the development of heart, vascular, and colon cancers (Divorty et al., 2018). Furthermore, next-generation drugs, such as small molecule inhibitors, might target this ligand–receptor axis to alter GPR35^+^ cellular responses in the setting of AAV. For more accurate targeted therapy in the future, it is one of the most attractive therapeutic directions for AAV and can effectively aid in further clarifying the signaling pathways linked to MAPK and PI3K activation, and thus, open novel avenues in AAV treatment by intervening protein activity in these signaling pathways.

Theoretically, for rapidly reducing the remission of autoimmune diseases, PLEX therapy can rapidly remove the immune active molecules in the patient, including autoantibodies and various proinflammatory cytokines. A few studies have shown that PLEX treatment is effective for acute renal damage of AAV (Walsh et al., 2011). In 2016, the European League Against Rheumatism (EULAR) recommended the treatment of life-threatening complications (severe diffuse alveolar hemorrhage and rapidly progressive glomerulonephritis) due to active AAV (Yates et al., 2016). However, a recent study has indicated that PLEX could not significantly reduce the incidence of death or end-stage renal disease (Walsh et al., 2020), which has led to clinical controversy (De Vriese and Fervenza 2021). Therefore, the therapeutic value of PLEX therapy in AAV needs to be carefully evaluated by using more extensive and rigorous clinical studies in the future.

## 8 Conclusion

Accumulating pieces of evidence have clearly demonstrated the important role of neutrophils in the pathogenesis and clinical damage of AAV. In AAV pathogenesis, neutrophil-related innate and adaptive immunity can function to promote the actions of each other. The various proteins in neutrophil cytoplasmic granules, such as MPO and PR3, contribute to producing MPO-ANCA and PR3-ANCA by activating adaptive immunity. These autoantibodies can exhibit multiple pathogenic effects. At the same time, the neutrophils can lead to AAV onset and cause clinical damage by activating ACP to produce C5a, expressing C5aR, producing NETs, exerting respiratory burst, and promoting degranulation. Many important signaling pathways have been reported to be involved in the regulation of neutrophil activity and function during AAV processes. Hence, with further understanding of neutrophils in the pathogenesis of AAV, novel targeted therapeutic have been developed, including Avacopan and RTX. In addition, some immune factors have been reported to be valuable for both the diagnosis and prognosis of AAV. Overall, an in-depth study on the potential role of neutrophils in AAV pathogenesis can lead to novel ideas and discoveries, which will contribute to better prognosis and longer life expectancy for AAV patients.

## Glossary

5-HIAA: 5-hydroxyin doleacetic acid
AAV: antineutrophil cytoplasmic antibody-associated vasculitis
ACP: alternative complement pathway
ANCA: antineutrophil cytoplasmic antibody
AZP: azathioprine
BAFF: B-cell activation factor
C5a: fragment a of fifth complement
C5aR: C5a receptor
CSS: Churg–Strauss syndrome
CTX: Cyclophosphamide
DNase: deoxyribonuclease
ECP: eosinophil cationic protein
EDN: eosinophil-derived neurotoxin
EGPA: eosinophilic granulomatosis with polyangiitis
ENT: ears, nose, and throat
EPO: eosinophil peroxidase
ERK: extracellular signal-regulated kinase
EULAR: European League Against Rheumatism
FDA: Food and Drug Administration
GC: glucocorticoids
G-CSF: granulocyte colony stimulating factor
GM-CSF: granulocyte macrophage colony stimulating factor
GPA: granulomatosis with polyangiitis
GPR: G-protein-coupled receptor
HES: hyper-eosinophilic syndromes
HMGB1: high-mobility group box 1
HSP27: heat-shock protein 27
IFN-α: interferon-α
IL-1: interleukin-1
LAMP-2: lysosome-associated membrane protein-2
MAP: mean airway pressure
MAPK: mitogen-activated protein kinases
MBP: major basic protein
MIF: macrophage migration inhibitory factor
MMF: mycophenolate mofetil
MPA: microscopic polyangiitis
MPO: myeloperoxidase
MTX: methotrexate
NE: neutrophil elastase
NET: sneutrophil extracellular traps
NOX: NADPH oxidase
PAK1: p21 activated kinase 1
PDK1: phosphoinositide-dependent kinase-1
PDK2: phosphoinositide-dependent kinase-2
PI3K: phosphoinositide 3-kinase
PIP2: phosphatidylinositol 4,5-bisphosphate
PIP3: phosphatidylinositol-3,4,5-triphosphate
PIP4: phosphatidylinositol-4-diphosphate
PLEX: plasma exchange
PMN: polymorphonuclear neutrophils
PR3: proteinase 3
Rac1: Ras-related C3 botulinum toxin substrate 1
RTX: Rituximab
T308: threonine 308
TFH: T follicular helper cells
TLRs: Toll-like receptors
TNF-α: tumor necrosis factor-α
WG: Wegener granulomatosis
